# One pot facile transformation of CO_2_ to an unusual 3-D nano-scaffold morphology of carbon

**DOI:** 10.1038/s41598-020-78258-6

**Published:** 2020-12-09

**Authors:** Xirui Wang, Gad Licht, Xinye Liu, Stuart Licht

**Affiliations:** Department of Chemistry, George Washington University, Washington, DC 20052 USA

**Keywords:** Chemistry, Engineering, Materials science, Nanoscience and technology, Climate change

## Abstract

An electrosynthesis is presented to transform CO_2_ into an unusual nano and micron dimensioned morphology of carbon, termed Carbon Nano-Scaffold (CNS) with wide a range of high surface area graphene potential usages including batteries, supercapacitors, compression devices, electromagnetic wave shielding and sensors. Current CNS value is over $323 per milligram. The morphology consists of a series of asymmetric 20 to 100 nm thick flat multilayer graphene platelets 2 to 20 µm long orthogonally oriented in a 3D neoplasticism-like geometry, and appears distinct from the honeycomb, foam, or balsa wood cell structures previously attributed to carbon scaffolds. The CNS synthesis splits CO_2_ by electrolysis in molten carbonate and has a carbon negative footprint. It is observed that transition metal nucleated, high yield growth of carbon nanotubes (CNTs) is inhibited in electrolytes containing over 50 wt% of sodium or 30 wt% of potassium carbonate, or at electrolysis temperatures less than 700 °C. Here, it is found that a lower temperature of synthesis, lower concentrations of lithium carbonate, and higher current density promotes CNS growth while suppressing CNT growth. Electrolyte conditions of 50 wt% sodium carbonate relative to lithium carbonate at an electrolysis temperature of 670 °C produced over 80% of the CNS desired product at 85% faradaic efficiency with a Muntz brass cathode and an Inconel anode.

## Introduction

Highly porous, nano-thick extended 3D carbon morphologies termed carbon scaffold have been of interest since at least 1994^1^, and have been commercialized^2^. Such morphologies possess exceptional strength to weight ratio, super-elasticity during repeated compression cycles, and have the high surface area and material compatibility for applications such as high capacity battery anodes, supercapacitors, high-efficiency electromagnetic wave absorption, and sensors. There have been a variety of studies^2–10^ and simulations^11^ reported of such 3-D multilayered graphene morphologies. Often, these structures are synthesized by Chemical Vapor Deposition (CVD) onto an existing metal carbon nanofoam, followed by acid etch of the metal, and leaving only the residual Graphene Foam (GF) with the structure of the original metal foam template. Nickel and copper nano-templates have been used in their preparation^2–4^. In a modification of this methodology for battery^5^ and supercapacitor^9^ applications this metal foam can be left in-place. Another GF synthesis methodology is via thermal expansion at 1000 °C of pyrolytic graphite containing intercalated chloroaluminate ions leading to a 120-fold expansion^6^, or similarly by intercalation of sulfuric acid into expanded graphite^8^. Additionally, such foams have been synthesized by freeze drying a solution of graphene oxide and subsequent annealing in an argon atmosphere^9,10^. Finally, a series of these carbon scaffold morphologies have been formed by carbonizing organic foams or wood with a typical balsa or balsa like cellular configuration. In each case the foams or scaffolds assume a 3D grid comprised of a range of spherical or crater-like cells^12–20^. However, these methods to produce high-quality GF costs over $223 per milligram of material making them impractical for large scale usage, often are energy intensive, and use hydrocarbons that contribute to greenhouse gas emissions^2,21^. Therefore, it is necessary if large scale usage of graphene nanofoams is to occur, to discover and report a green, inexpensive production methodology.

Here we present synthesis of an unusual rectangular scaffold morphology, a Carbon Nano-Scaffold (CNS) comprised of nano-thick graphene flakes in a neoplasticism abstract art-like arrangement. The synthesis is unusual not only in the morphology of the product and that it is spontaneously formed without a template in a one-pot synthesis, but also in its green carbon negative footprint. The concentration of the greenhouse CO_2_ in the atmosphere, which had cycled around 235 ± ∼ 50 ppm for 400,000 years millennia until 185, is currently at 416 ppm and rising at a rapid annual rate incurring planetary climate disruptions, global warming and habitat loss^22–25^. Transformation of CO_2_ into a non-greenhouse gas posed a major challenge as it was regarded as such a stable molecule^26^. The conventional methodology of carbon nanomaterial production is CVD that has a high carbon footprint; CVD is an expensive process, energy intensive and is associated with an unusually massive carbon footprint of up to 600 tonnes of CO_2_ emitted per tonne of carbon nanomaterial produced^21^. Rather than emitting CO_2_, this new electrosynthetic methodology uses CO_2_ as a reactant and is carbon negative. This is especially true if one uses renewable energy as source of electricity; however, even with fossil fuels energy this may be true^27–29^. This is because all fossil fuels, with exception of coal, get energy from hydrogen and carbon combustion with extra energy form burning hydrogen part of hydrocarbon leading to some cases a net negative carbon footprint as seen for some CNT production we have done in the past (energy produced by burning fuel provides more energy than needed to turn CO_2_ into product, that is excess electricity is produced in addition to the CNT product)^27,28^. This may even be furthered if it reduces amount of other high emitting CO_2_ materials needed^29^.

In 2010, we demonstrated the efficient splitting of CO_2_ in a molten Li_2_CO_3_ electrolyte into both pure solid carbon at 750 °C and CO at 950 °C using renewable energy^30,31^. Molten lithium carbonate has a high affinity for CO_2_, and molten carbonate electrolysis offers a novel, efficient methodology for the CO_2_ transformation to a variety of useful products including syngas and methane^32–34^. In 2015, we noted the solid carbon product of CO_2_ electrolysis in molten carbonate can be a high yield of Carbon Nanotubes (CNTs) through the addition of transition metals including Ni, and then Cr and others acting as nucleation points^35^. We termed this new synthetic route to CNTs the Carbon dioxide to Carbon NanoTubes (C2CNT) process^27–29,35–44^, and noted that these CNTs can be useful to massively reduce the carbon footprint of high strength composite structural materials^29^. In the C2CNT process, we have demonstrated and quantified the affinity of molten carbonates to absorb both atmospheric and flue gas levels of CO_2_, and have utilized ^13^C isotope CO_2_ to track^37^ and demonstrate in molten lithium carbonate that CO_2_ originating from the gas phase serves as the renewable carbon building blocks in the observed CNT product and the net reaction is in accord with:

1 $${\text{Dissolution}}:{\text{ CO}}_{{2}} \left( {{\text{gas}}} \right)\, + \,{\text{Li}}_{{2}} {\text{O}}\left( {{\text{soluble}},{\text{ produced by electrolysis}}} \right)\, \rightleftharpoons \,{\text{Li}}_{{2}} {\text{CO}}_{{3}} \left( {{\text{molten}}} \right)$$

2 $${\text{Electrolysis}}:{\text{ Li}}_{{2}} {\text{CO}}_{{3}} \left( {{\text{molten}}} \right)\, \to \,{\text{C}}\left( {{\text{CNT}}} \right)\, + \,{\text{Li}}_{{2}} {\text{O }}\left( {{\text{soluble}}} \right)\, + \,{\text{O}}_{{2}} \left( {{\text{gas}}} \right)$$

3 $${\text{Net}}:{\text{ CO}}_{{2}} \left( {{\text{gas}}} \right)\, \to \,{\text{C}}\left( {{\text{CNT}}} \right)\, + \,{\text{O}}_{{2}} \left( {{\text{gas}}} \right)$$

This process below 800 C does not generate CO as previous experiments and calculations so that CO is only formed due to energetics at temperature above 800 °C^30,31^.

**Figure 1:**
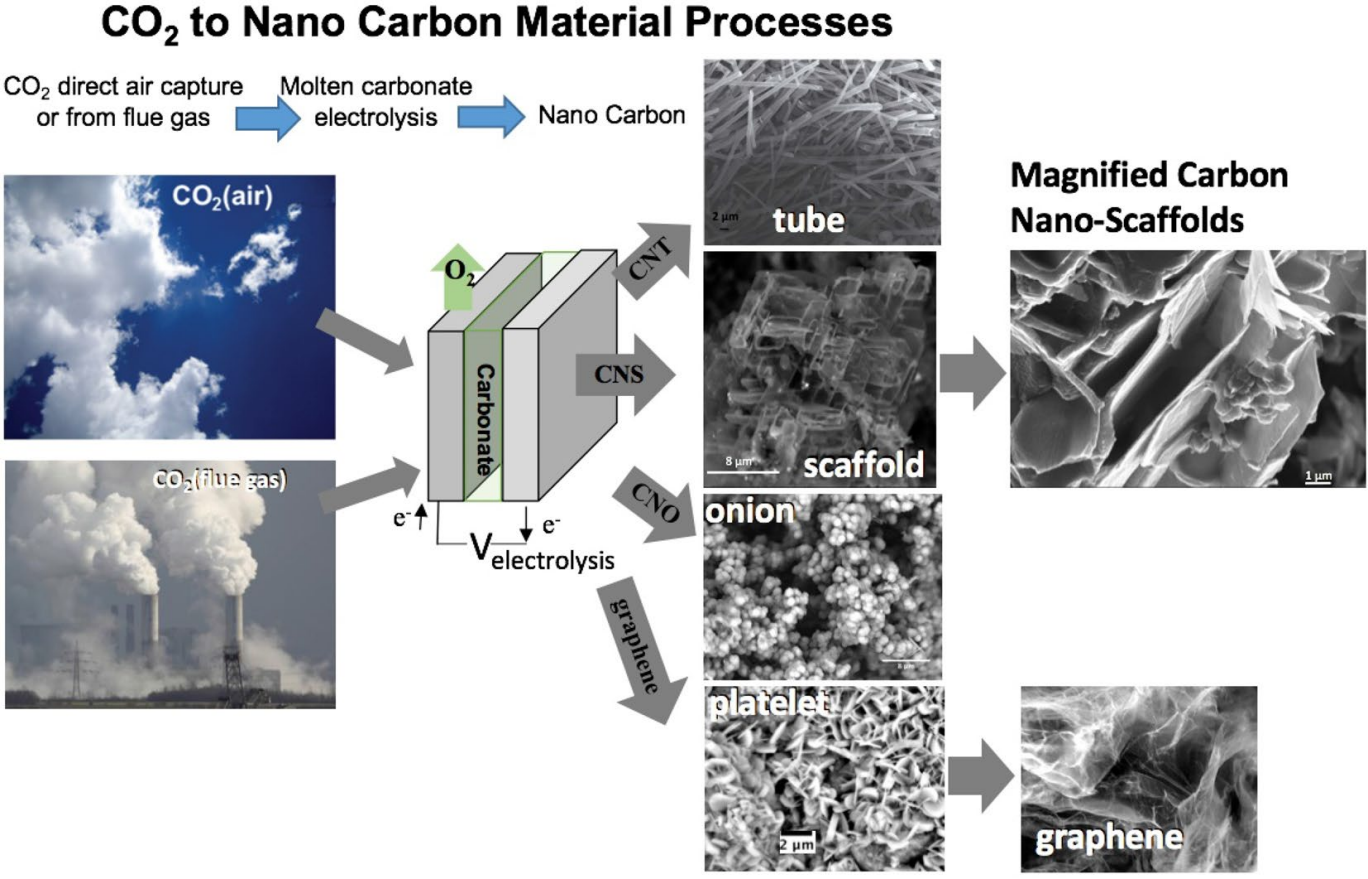
High yield electrolytic synthesis of carbon nano materials from CO_2_, either directly from the air or from smoke stack CO_2_, in molten carbonate.

An important component of the C2CNT growth process is transition metal nucleated growth of the CNTs. The nucleation occurs with metal migration from the anode as it establishes a stable oxide electrocatalytic anodic overlayer, or by the simple addition of metal oxides or metal powders to the electrolyte or cathode surface. In the absence of transition metal nucleating agents, CNTs are scarce, comprising < 1% of the carbon product. However, as illustrated in Fig. 1, in the absence of transition metal nucleating agents, we have recently demonstrated that other uniform products, Carbon Nano-Onions (CNOs), Carbon Nano-Platelets (CNPs) and graphene can be synthesized instead via molten carbonate electrolysis of CO_2_^45–48^.

4 $${\text{CO}}_{{2}} \left( {{\text{gas}}} \right)\, \to \,{\text{C}}\left( {{\text{CNO}}} \right)\, + \,{\text{O}}_{{2}} \left( {{\text{gas}}} \right)$$

5 $${\text{CO}}_{{2}} \left( {{\text{gas}}} \right)\, \to \,{\text{C}}\left( {{\text{CNP}}} \right)\, + \,{\text{O}}_{{2}} \left( {{\text{gas}}} \right)$$

6 $${\text{CO}}_{{2}} \left( {{\text{gas}}} \right)\, \to \,{\text{C}}\left( {{\text{graphene}}} \right)\, + \,{\text{O}}_{{2}} \left( {{\text{gas}}} \right)$$

In this new study, we demonstrate a pathway to synthesize an additional, unique, uniform carbon morphology product, carbon nano-scaffolds CNS, as illustrated on the right, middle of the Fig. 1.

7 $${\text{CO}}_{{2}} \left( {{\text{gas}}} \right)\, \to \,{\text{C}}\left( {{\text{CNS}}} \right)\, + \,{\text{O}}_{{2}} \left( {{\text{gas}}} \right)$$

As described here the successful molten carbonate CO_2_ electrolysis to the unusual morphology, new CNS product occurs by conducting the electrolysis in electrolytic conditions not conducive to CNT formation (inhibiting nucleation points) consisting of electrolytes with decreased lithium concentration, and either at lower temperatures or at higher electrolysis current density. This new method provides a novel, inexpensive method to remove CO_2_ and potentially turn it from a greenhouse gas pollutant into a useful, unusual carbon morphology product.

## Experimental

### Experiment materials and procedure

Lithium carbonate (Li_2_CO_3_, 99.0%, Rockwood Lithium), sodium carbonate (Na_2_CO_3_, 99.98%, Sigma Aldrich), potassium carbonate (K_2_CO_3_, 99.95% Sigma Aldrich), boric acid (H_3_BO_3_) and anhydrous lithium metaborate (LiBO_2_, 99.9%, Alfa Aesar) are used as the electrolyte in this study. The electrolyte is pre-mixed in the noted ratios. The 0.25-inch-thick Muntz brass sheet used as the cathode, and the 0.05-inch-thick Inconel 718 (both purchased from onlinemetal.com) is used as the anode^44^.

### Electrolysis and purification

Electrodes are perpendicularly immersed into the freshly made molten salt electrolyte with a 1 cm separation. The electrolyte and electrodes are contained in a rectangular stainless steel 304 case or an alumina crucible (Fig. 7) and electrolyses conducted under heated ambient air (~ 416 ppm CO_2_). Electrodes of electrolyses in Fig. 4 are as described in references 45 and 46. All other electrolyses are conducted with Muntz brass cathodes (an alloy of 60 wt% Cu and 40 wt% Zn) and anodes as described in the main text. Electrodes are reusable and electrolyses are conducted under conditions of constant current density. The raw product is collected from the cathode after the experiment and cool down, followed by an aqueous HCl wash procedure. As previously described^45^. The washed carbon product is separated by vacuum filtration. The washed carbon product is dried overnight at 60 °C oven yielding a black power product. The synthesis yield (Coulombic efficiency) is calculated in Eq. (8) as described previously^44^:

8 $$\text{{Coulombic}}\, \text{{efficiency}}=100\mathrm{\% }\times \frac{{C}_{experimental}}{{C}_{theroretical}}$$

The *C*_*experimental*_ is determined by the dried final product mentioned in the previous section, and the following Eq. (9) calculates the *C*_*theoretical*_:

9 $${C}_{theoretical}=\frac{Q}{nF}\times 12.01g\cdot C{\cdot mol}^{-1}{\cdot e}^{-}$$

where *Q* is the total electrolysis charged applied, *F* is the Faraday (96485A*s*mol^−1^ e^−^), and the *n* is the value of electron for the reduction of tetravalent carbon, 4e^−^ mol^−1^.

### Characterization

Samples are analyzed by PHENOM Pro Pro-X SEM, or FEI Teneo LV SEM, FEI Helios FIB SEM, and by FEI Teneo Talos F200XTEM as previously described^44^. Raman spectra were collected with a LabRAM HR800 Raman microscope (HORIBA). This Raman spectrometer/microscope uses an incident laser light with a high resolution of 0.6 cm^−1^ at a 532.14 nm wavelength. TGA were conducted with a TA 2950 Analyzer with temperature ramped at 60 °C per hour. XRD was measured with a Rigaku D = Max 2200 XRD.

## Results and discussion

We have consistently observed that both lithium carbonate enriched electrolytes and transition metal nucleation points facilitate CNT growth during the electrolysis of CO_2_ in molten carbonates^27–29,35–43^. When transition metal nucleation sites are inhibited from formation during the synthesis, for example by replacing the nickel containing anode with a more noble metal such as iridium, platinum, or a noble metal alloy, we have then observed and demonstrated the high yield electrosynthesis of other carbon nanomaterials from electrolyses in Li_2_CO_3_ electrolyte, such as carbon nano-onions, nano-platelets and graphene (as shown in Fig. 1)^45–47^.

In this study, three other electrolysis conditions are shown here to inhibit transition metal nucleation and promote growth of alternative morphologies than CNTs, carbon nano-onions or platelets. The conditions are (1) a decrease in the electrolysis temperature, (2) a decrease in the concentration of lithium in the molten carbonate electrolyte, and with decreased lithium concentration even at higher temperatures, conditions of (3) higher electrolysis current density.

**Figure 2:**
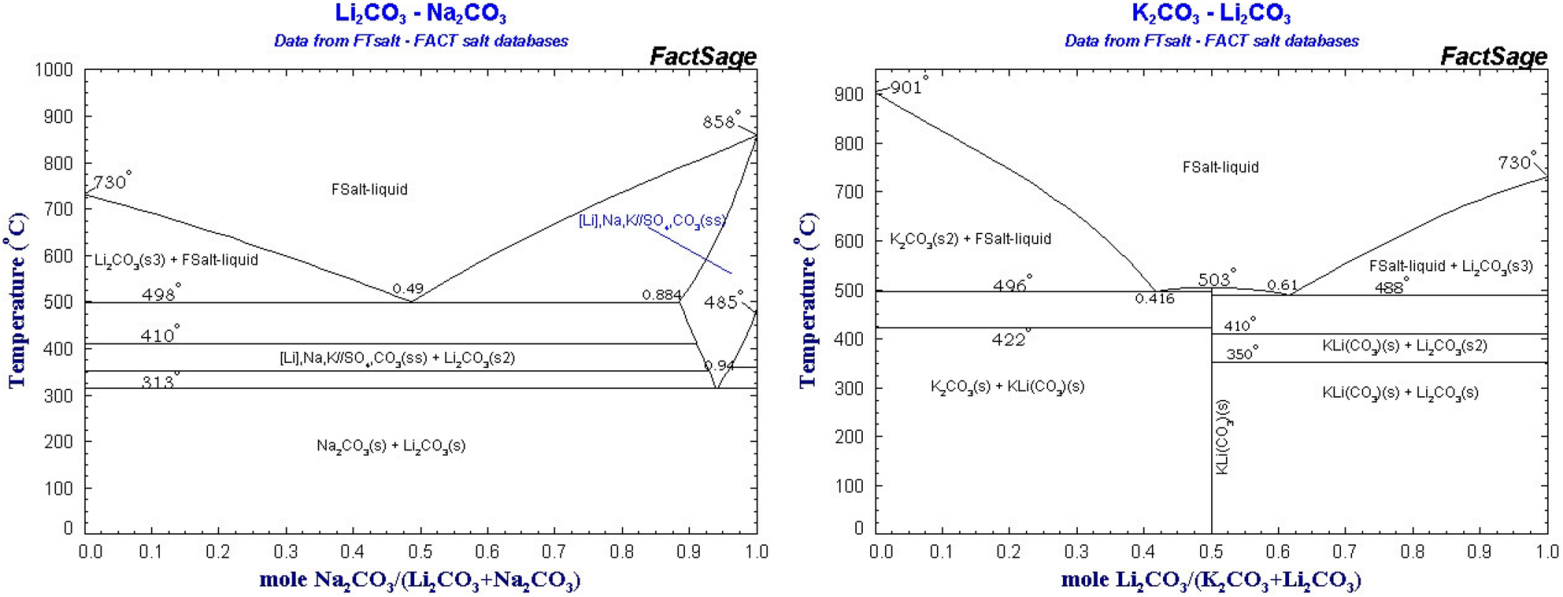
Phase diagrams of Li_2_CO_3_/Na_2_CO_3_/ and K_2_CO_3_/Li_2_CO_3_ binary mixtures as downloaded and redrawn from the FTsalt ^.^ FACT salt database.

Pure Li_2_CO_3_ melts at 730 °C, Na_2_CO_3_ melts at 858 °C, and K_2_CO_3_ melts at 901 °C. Consistent with eutectic mixtures, binary mixtures of lithium carbonate with these other carbonates melt at lower temperature than either of the pure components comprising the binary mix. Figure 2 presents the phase diagram of Li_2_CO_3_/Na_2_CO_3_/ and K_2_CO_3_/Li_2_CO_3_ binary mixtures from the FTsalt FACT salt database. It is seen that melting points as lower than 500 °C can be achieved with appropriate concentrations of either binary mixture.

We have shown that temperatures greater than 700° are more conducive to CNT growth during molten carbonate electrolysis^40^. Here, it is also demonstrated that electrolytes with an increasing fraction of Na_2_CO_3_ or K_2_CO_3_ in a mixed Li_2_CO_3_ electrolysis are less conducive to CNT growth even in the presence of nucleating transition metals.

Li_2_CO_3_ melts above 700 °C, but as evident in Fig. 2 binary carbonates melt at lower temperatures allowing access to a lower temperature domain to explore CO_2_ electrolysis in molten carbonates. We have previously noted that lower than a 700 °C electrolysis temperatures inhibits CNT formation^40^, and thought it would be of interest to explore the morphology of a CO_2_ electrolysis product in which CNT formation is suppressed due to the dual effects of high sodium concentration and lowered temperature. Electrolyses of the 50% Na_2_CO_3_ / 50% Li_2_CO_3_ by weight electrolyte were conducted at 670 °C, rather than above the 730 °C melting point of pure Li_2_CO_3_.

**Figure 3:**
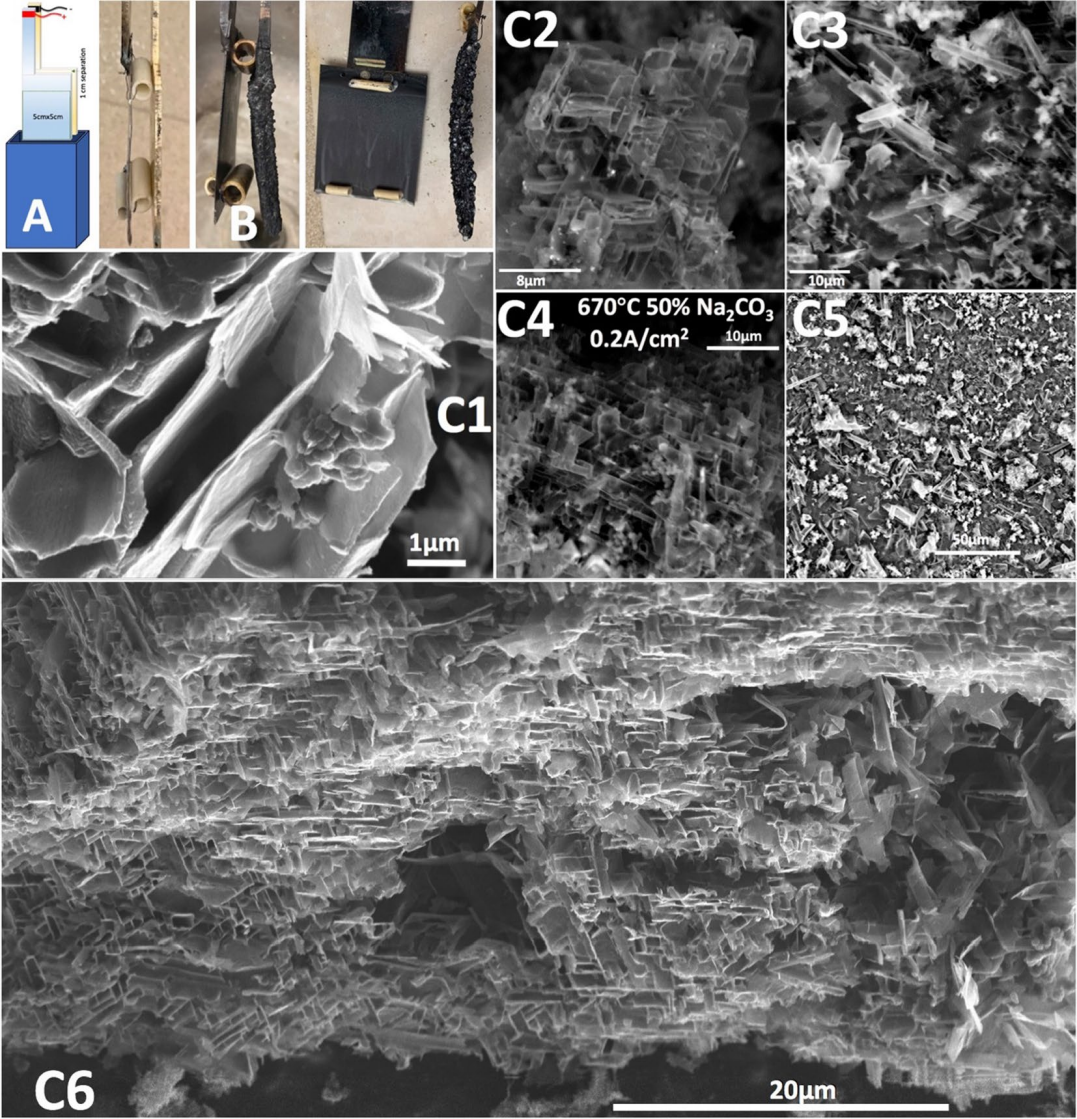
The unusual product of electrolysis at 670 °C in a mixed 50% Li_2_CO_3_/50% Na_2_CO_3_ relative wt% electrolyte: carbon nano-scaffolds. A scheme of the electrolysis cell is shown in A, and in B are shown the 5 cm x 5 cm Muntz brass cathode and Inconel 718 anode electrodes before and after electrolysis. The electrolyte consisted of 250 g of Na_2_CO_3_, 250 g of Li_2_CO_3_, and 50 g of H_3_BO_3_. The electrolysis was conducted at a current density of 0.2 A/cm^2^ at 670 °C for 4.0 h.

Figure 3 presents the unexpected, distinctive carbon nano-scaffold morphology product when the electrolysis is conducted at 670 °C. Over 80% of the product is this unusual carbon nano-scaffold morphology. Other product formed were graphitic, ball-like carbon, or amorphous carbon. As we have previously reported the 2e^−^ reduction of CO_2_ to CO, rather than the 4e^−^ reduction to solid carbon products is only observed at temperatures above 800 °C and becomes the dominant process at temperatures above 900 °C^31,33^. The only gas produced is O_2_ in a stoichiometric concentration relative to the the carbon produced during the electrolysis of CO_2_^44^. As evident in the highest magnification SEM in Fig. 3-C1, the morphology consists of a series of asymmetric 20 to 100 nm thick flat multilayer graphene platelets 2 to 20 μm long oriented in a 3D neoplasticism-like geometry. The morphology appears distinct from the honeycomb, foam, or balsa wood cell structures previously attributed to carbon. Although this appears morphologically distinct from those previous structures, CNS also describes this morphology. While transition metal elements can again be release from the Inconel 718 used as the anode, and while the Muntz brass cathode is comprised of copper and zinc, there is no evidence that the observed carbon nano-scaffold product is based transition metal nucleation as probed through SEM, EDS and TEM^35^. Voltage throughout the electrolysis was consistently 2.0 V, and over 85% of theoretically (from the 4e^−^ reduction) calculated transformation of as measured by Eq. (8) of the CO_2_ was converted to carbon. This coulombic (current) efficiency to produce the carbon nanoscaffolds is lower than the 98–100% typically observed in the molten carbonate electrosynthesis of carbon nanotubes, but the latter process has undergone considerably more optimization of the individual controlling electrochemical parameters.

**Figure 4:**
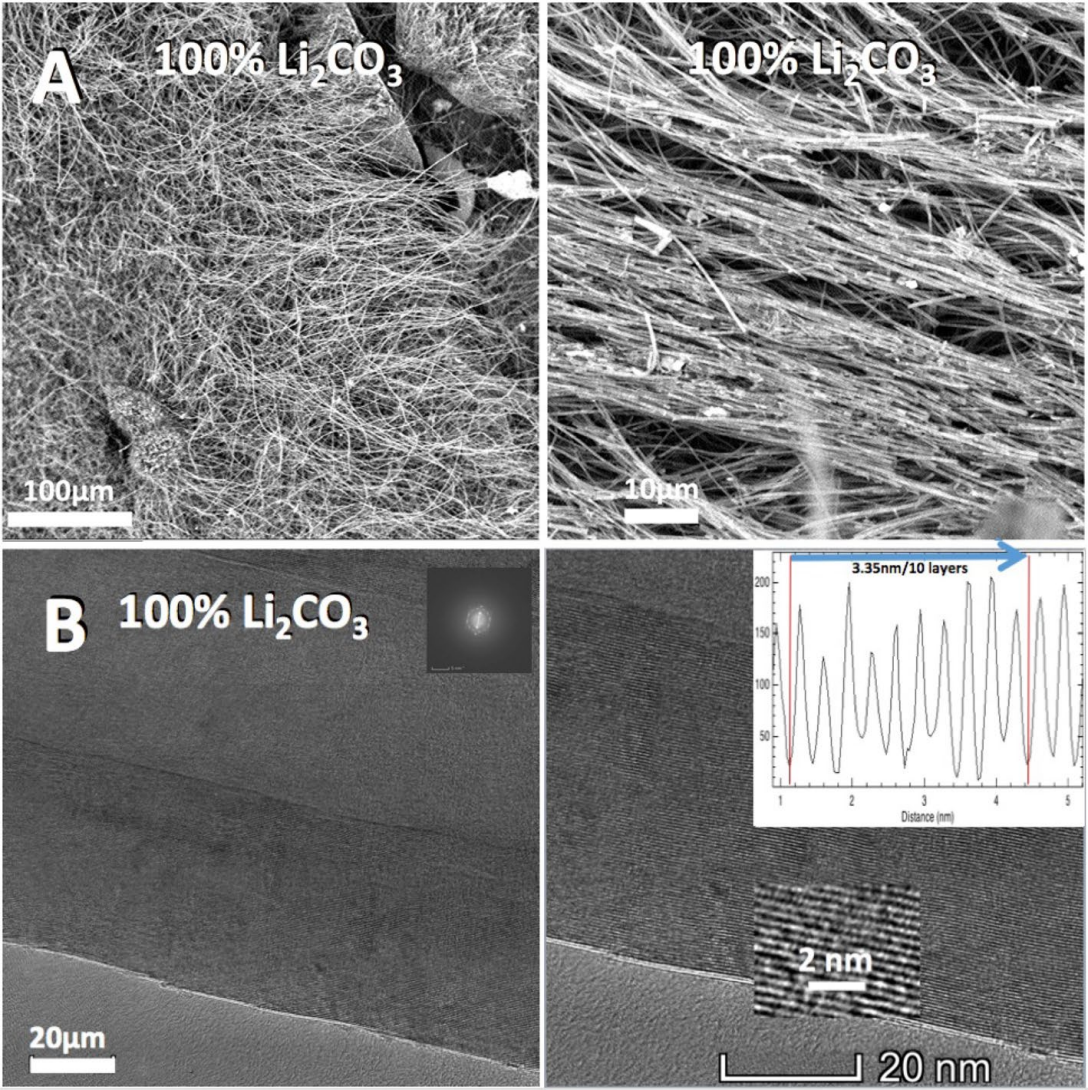
A SEM and B TEM of the high yield CNT product from electrolysis in pure Li_2_CO_3_ electrolyte at 770 °C at a constant current density of 0.2 A/cm^2^. (**A**) (SEM) & (**B**) (TEM) of the high yield CNT product.

**Figure 5:**
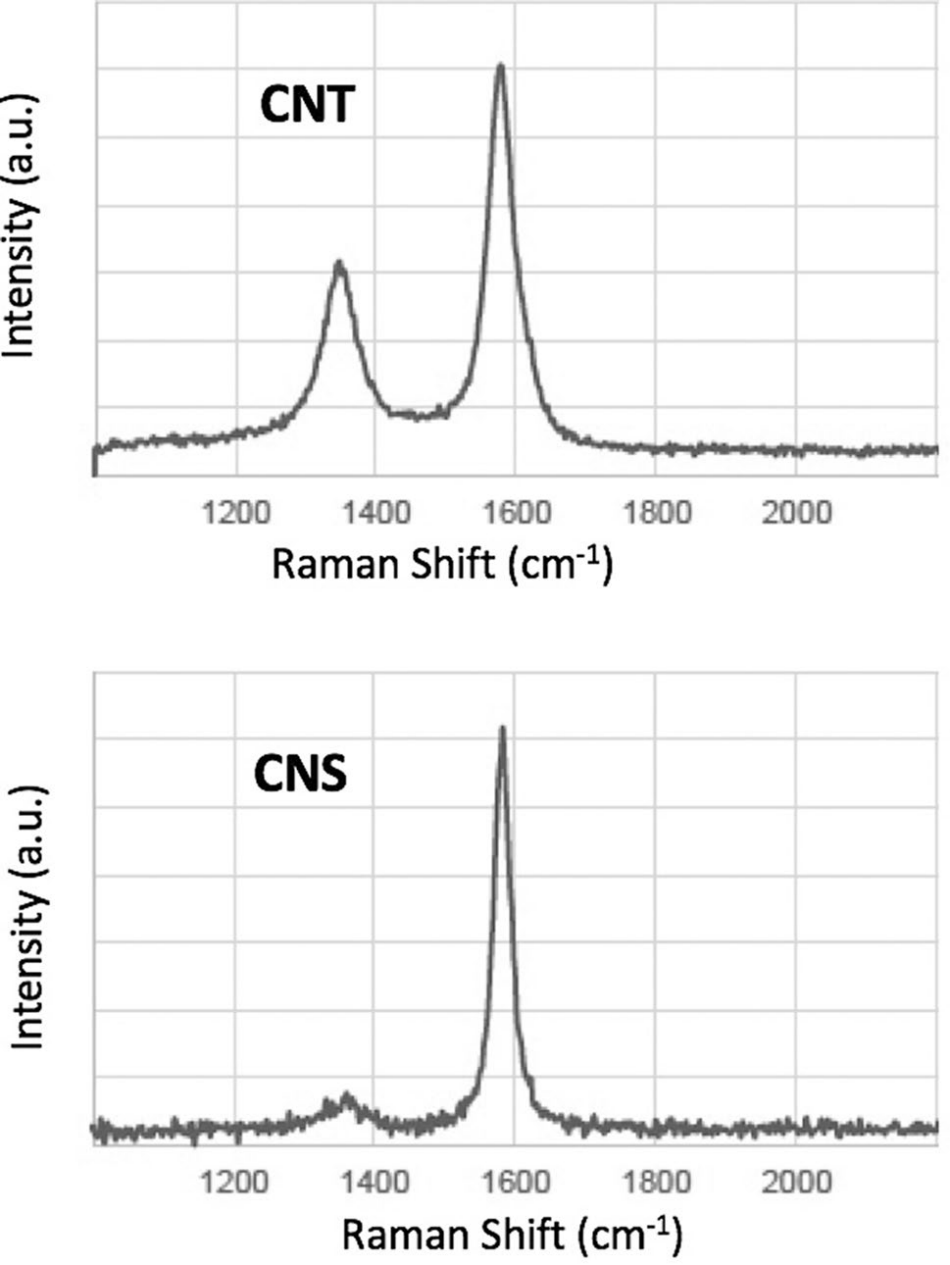
Raman spectra of the CNT (top) and CNS (bottom) products.

During molten carbonate electrolysis of CO_2_, transition metal nucleated growth of CNT at the cathode is promoted with the use of a high stable nickel alloys^44^, including Inconels, Nichromes (Chromels) and Ni C276, which release both Ni and Cr to the electrolyte as it forms a stable oxide layer for oxygen generation during the electrolysis. This study purposely inhibits CNT nucleation to probe which other carbon nanomaterial morphologies may be grown instead. As shown in Fig. 4A, the product from electrolysis in pure lithium carbonate is uniform, highly pure CNTs. The addition of nickel powder, or migration of nickel leads to clearly observable CNT walls as shown in Fig. 4B, with concentric walls separated by 0.335 nm, and typica1 of the distinctive one atom thick separation of multiple graphene layers. TGA of CNT product of the pure Li_2_CO_3_ electrolyte synthesis exhibits no oxidation through 450 °C, 10 wt% oxidation of the product by 560 °C, half the product oxidized by 615 °C, and oxidation complete by 700 °C. XRD of both the CNS product and the CNT product exhibits a sharp peak at 2 = 26.3° indicative of a high degree of graphitic allotrope crystallinity. TGA of the Fig. 3 CNS synthesis product exhibits no oxidation through 430 °C, 10 wt% oxidation of the product by 500 °C, half the product oxidized by 550 °C, and oxidation complete by 660 °C. This compares with amorphous carbon which starts to oxidize at ~ 300 °C, and provides evidence the carbon scaffold structure has oxidation resistance intermediate to that of coal and CNTs and with its graphitic structure has a combustion resistance closer to that of CNTs. Raman spectrum of the CNT and CNS products exhibits two peaks at 1350 cm^−1^ and 1575 cm^−1^, which correspond to the disorder-induced mode (D band) and the high frequency E2g first order mode (G band) peaks respectively. Interestingly, as shown in Fig. 5, the CNS product displays a small I_D_/I_G_ peak ratio indicative of less disorder, that is more sp^2^ bonding and fewer sp^3^ defects, indicative that the CNS product is more representative of a graphene-like morphology than the CNT product. The observed I_D_/I_G_ for the CNT is considered representative of that for multiwalled carbon nanotubes.

**Figure 6:**
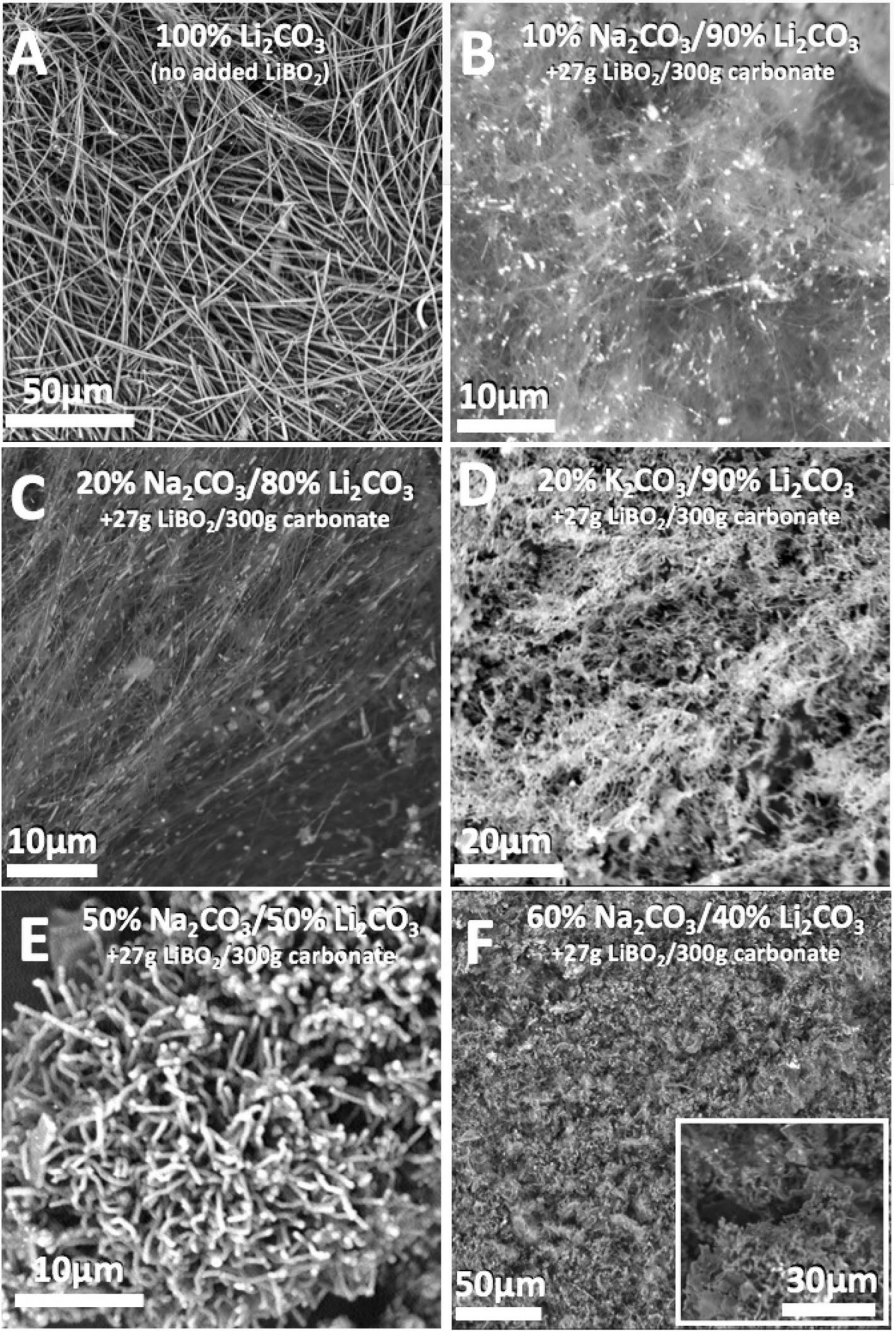
SEM of the CNT product from 770 °C electrolyses in mixed Li_2_CO_3_ electrolyte containing Na_2_CO_3_ or K_2_CO_3_ at the relative wt% shown on the figure panel at a current density of 0.2 A/cm^2^ with a cathode of Muntz and an anode of Inconel.

Figure 6B–F shows SEM of the electrolysis product occurring in various mixed electrolytes compared to that in panel 6A which is conducted in a pure, 24 h aged, 770 °C Li_2_CO_3_ electrolyte subsequent to a 5 h electrolysis. The SEM in Fig. 6A,F are at both with a scale bar of 50 µm, and the comparison is striking in that there are no CNTs readily observed in the 60% Na_2_CO_3_/40% by weight Li_2_CO_3_ electrolysis product, while the product is highly uniform CNTs form the 100% Li_2_CO_3_ electrolysis. Each of the electrolyses is conducted at a current density of 0.2 A/cm^2^ with a cathode of Muntz Brass (an alloy of 60% Cu and 40% Zn) and an anode of Inconel 718 (an alloy of 50–55% Ni, 17–21% Cr, 2, 4.75–5.5% Nb & Ta, 2.8–3.3% Mo, the remainder Fe with low concentrations of Ti, Co, Al, Mn, Cu, Si and C). The addition of 8% LiBO_2_ to the electrolyte further improves the morphology, uniformity and purity of the CNT product. For example, addition of 8% LiBO_2_ to the pure Li_2_CO_3_ increased the aspect ratio (length to diameter) of the CNT product (not shown), and this LiBO_2_ (or H_3_BO_3_) was added to each of the mixed electrolytes to improve the lower quality of the CNT product. H_3_BO_3_, rather than Li_2_BO, was added to some electrolytes as a cost saving measure. H_3_BO_3_ upon heating reacts with the carbonate salt releases water, and contributes to the electrolyte melt the same boron valence state oxide as Li_2_BO.

**Figure 7:**
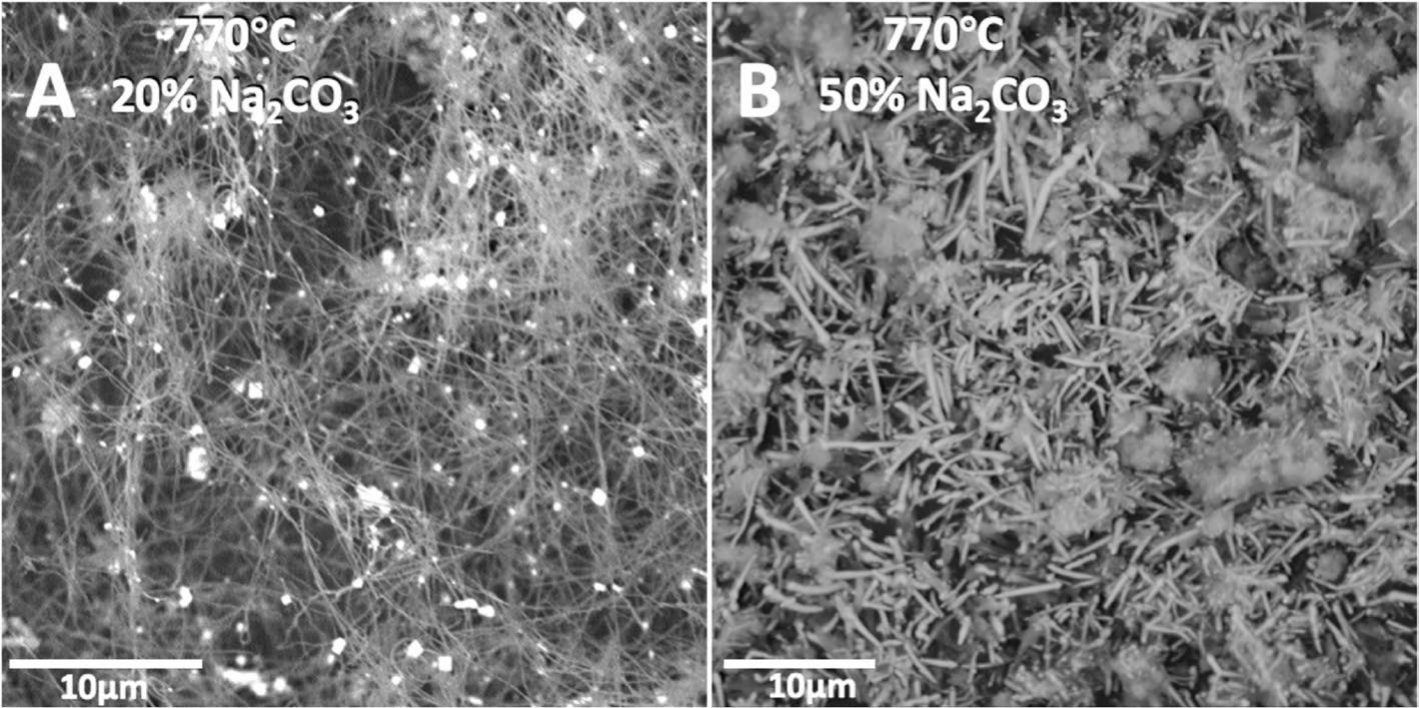
Mixed Li_2_CO_3_ electrolyte 770 °C electrolyses product; SEM of the washed carbon product using electrolytes containing either 20 wt% Na_2_CO_3_ (**A**) or 50% wt% Na_2_CO_3_ (**B**) relative to the Li_2_CO_3_. Electrolyses include an additional 10 wt% H_3_BO_3_ which promotes uniform morphology. Bright dots in SEM figures indicate the presence of transition metals, and are not seen elsewhere in later images.

When the electrolysis is conducted at the same current density and in a similar composition 50% Na_2_CO_3_/50% Li_2_CO_3_ electrolyte as employed at 670 °C in Fig. 2, but instead at 770 °C in Fig. 6E no carbon nano-scaffolds are observed. When the relative lithium carbonate concentration is decreased from 80% to 50 wt% in the mixed Na_2_CO_3_ electrolyte, the CNT product in Fig. 6E, lose the high aspect ratio evident in the 20% Na_2_CO_3_ electrolyte; that is the CNTs become short and thick, and are also less prevalent in the product. For the 60 wt% Na_2_CO_3_ electrolyte, as shown in Fig. 6F, the electrolysis product is composed of approximately half short CNTs, while the other half of the product is carbon platelets starting to arrange in a crude nano-scaffold morphology. In Fig. 6, the 10% or 20% Na_2_CO_3_ electrolysis products contain over 90% CNT. The 30% Na_2_CO_3_ (not shown), and 50% Na_2_CO_3_ (shown) exhibit a diminishing yield of CNTs accompanied by an increasing fraction of carbon nanospheres and carbon platelets in the product. The CNT diameter increases with increasing Na_2_CO_3_ percentage in the electrolyte (10 wt%: ~ 80 nm, 20 wt%: ~ 100 nm, 30 wt%: ~ 200 nm, 50 wt%: ~ 1 μm). For the 20 wt% K_2_CO_3_ in Li_2_CO_3_, SEM shown in Fig. 6D and 50 wt% K_2_CO_3_ (not shown) electrolyses, the loss of aspect ratio and the decrease in CNT purity occurs more rapidly with increasing K_2_CO_3_ weight fraction than in the electrosynthesis with increasing Na_2_CO_3_ fraction. An expanded view of the electrolysis product of similar 20 or 50 wt% Na_2_CO_3_ electrolytes is compared in Fig. 7. The electrolysis was conducted at 770 °C for 4.0 h at a constant current of 5 A with 5 by 5 cm electrodes with Muntz brass cathode and Inconel anode. 98.7% of the applied charge was converted to carbon in the 20% Na_2_CO_3_ electrolyte and that carbon consisted of over 90% CNTs. 93% of the applied charge was converted to carbon in the 50% Na_2_CO_3_ electrolyte, and 80% of that product was CNTs.

Energy-dispersive X-ray spectroscopy (EDS) tests were employed to probe the elemental analysis of products from the mixed electrolyte electrolyses. EDS of both the 20% Na_2_CO_3_ and 20% K_2_CO_3_ samples are 100% carbon, while the 50% Na_2_CO_3_ and 50% K_2_CO_3_ spectra are respectively 97.0% carbon and 97.8% carbon; boron in the CNTs is below the limits of EDS detection, while each exhibits the presence of small residual alkali (Na and K respectively) possibly due to insufficient rinsing of the product. The influence of the borate addition to the electrolyte on doping and increased electrical conductivity of the molten carbonate electrosynthesized carbon product was previously noted and will not be repeated here, and increasing with borate addition the extent of carbon in that product was measured at 0.7 to 2.0 atomic% by the shifts in the Raman 1580 cm^−1^ G peak^41^.

We have previously calculated that the thermodynamic potential for the reduction of the alkali carbonates decrease in the order E_K2CO_ > E_Na2CO3_ > E_Li2CO3_^31^. At higher voltage, an increasing concentration of either the K_2_CO_3_ or Na_2_CO_3_ salts would increase the possibility for reduction of the alkali cation to the alkali metal. This could occur, rather than the desired occurrence of the reduction of carbonate to carbon^44^. As described in the Experimental section, the coulombic efficiencies compare the mass of the product to the applied 4e^−^ per mole of charge. These coulombic efficiencies of the electrosynthesis approach 100% (98–100%) for the three cases of 10% Na_2_CO_3_, and 20% Na_2_CO_3_ and 100% Li_2_CO_3_ electrolyte experiments. Coulombic efficiency is high, but consistently, marginally decreases in binary lithium carbon electrolytes containing over 20% of sodium or potassium carbonate. For example, the coulombic electrolysis efficiency rises from 90% for the 60% Na_2_CO_3_ electrolyte to 93% for the 50% Na_2_CO_3_ electrolyte, to 95% for 30% Na_2_CO_3_ electrolyte. It is relevant to note that as we have previously shown, carbonate electrolysis is decreasingly conducive to a CNT product in electrolytes containing > 20 wt% Na_2_CO_3_ or ≥ 20 wt% K_2_CO_3_^44^.

**Figure 8:**
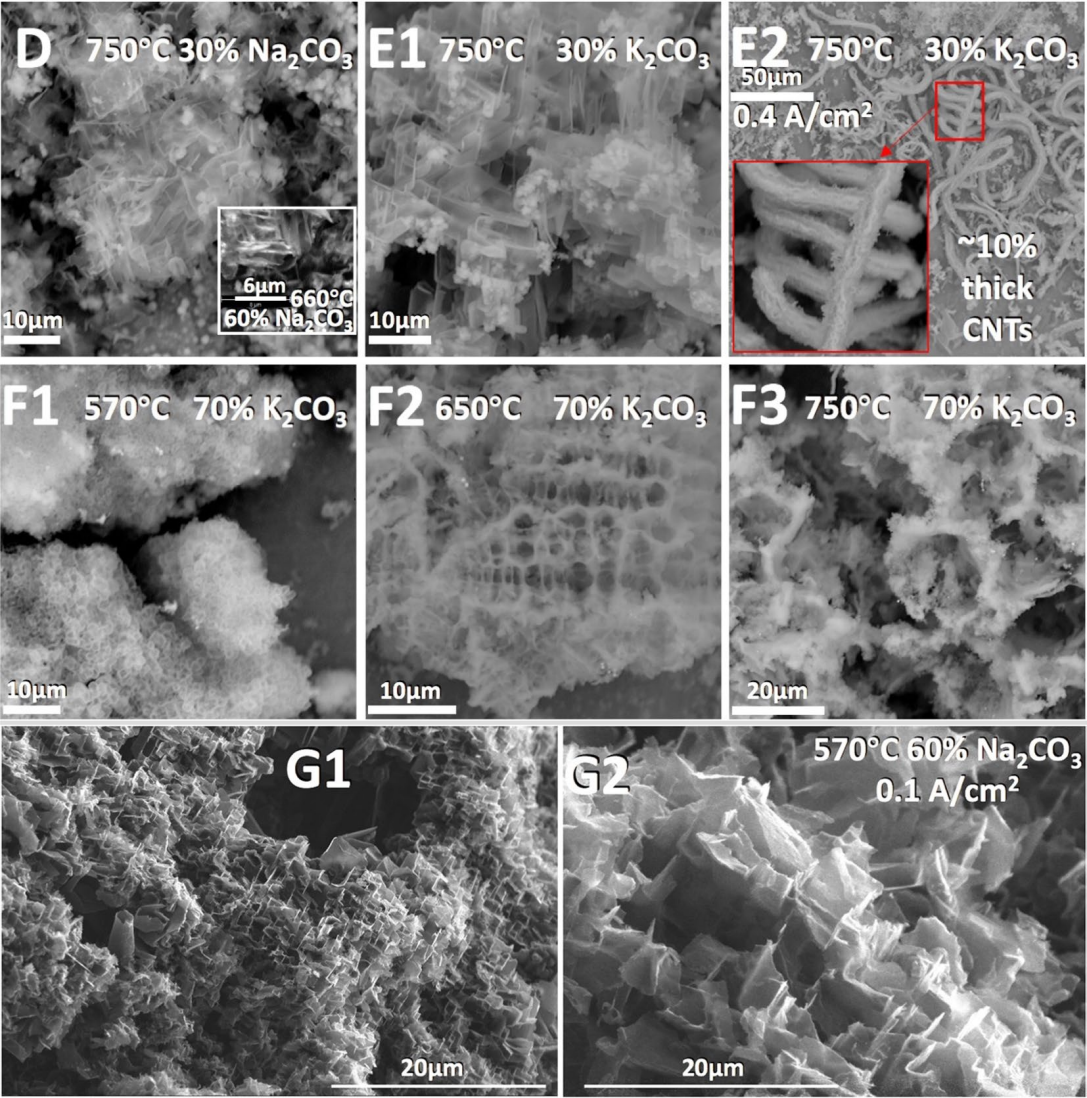
The unusual product of mixed binary alkali electrolytes, without added borate, with a different anode, Nichrome C, and the same cathode (Muntz brass). D and E: at high current density (0.4 A/cm^2^), and high T (other than inset) with various electrolytes. F: at high current density (0.4 A/cm^2^), at various T with a 70/30 wt% K_2_/Li_2_CO_3_ electrolyte. G: at low current density (0.1 A/cm^2^), low T, with a 60/40 wt% Na_2_/Li_2_CO_3_ electrolyte.

Figure 8 panels D through F, presents carbon products formed at a higher electrolysis current density (0.4 A/cm^2^), while panel G presents carbon products formed at a lower electrolysis current density (0.4 A/cm^2^), all with a different nickel anode then used previously, Nichrome C (61% Ni, 15% Cr, 24% Fe), the same cathode, and without any borate additive. Figure 8 panels D through F, probes carbon products formed over a range of temperatures. The top row compares the product at 750 °C and at a constant 30 wt% of either sodium or potassium carbonate as the binary component to lithium carbonate in the electrolytes. With the higher current density, as seen in panels D and E1 of Fig. 8, there is a significant carbon nano-scaffold product even at this higher temperature of 750 °C. The product of the 30% Na_2_CO_3_ electrolysis is large proportions of both carbon nano-scaffolds and carbon nano-onions. Not shown is that carbon nano-scaffolds are also observed in a 70 wt% Na_2_CO_3_ electrolyte, but the structures are smaller and are surrounded by amorphous carbon. At this temperature and current density, as seen in E-1 and E-2, the product of the 30% K_2_CO_3_ electrolysis consists mainly of carbon nano-scaffolds and ~ 10% very thick CNTs. EDS verifies that the carbon nano-scaffold structures are largely carbon (98.3%) with a small amount of residual potassium (1.7%). The carbon nano-scaffold is observed at 50 wt% K_2_CO_3_ (not shown), but is not observed in the electrolysis product from a 70 wt% K_2_CO_3_ electrolyte. The product of this 70 wt% K_2_CO_3_/30% Li_2_CO_3_ electrolyte is compared in the middle portion of Fig. 8 when the electrolysis is conducted at either 570 °C (F1), 650 °C (F2), or 750 °C (F3). In this electrolyte, at 570 °C the F1 product consists of small rounded, carbon assemblies, at 650 °C the F2 product consists of coral-like carbon assemblies, and at 750 °C the F3 panel product consists of larger, but less defined, coral-like carbon structures.

The inset of panel 7D shows that with the high current density of 0.4 A/cm^2^ in a 60/40 wt% Na_2_/Li_2_CO_3_ electrolyte, the nano-scaffold morphology is still observed when the temperature is decreased to 660 °C. Carbon nano-scaffolds can also synthesized at a low current density of 0.1 A/cm^2^ when the temperature is decreased further to 570 °C as shown in Fig. 8 panels G, although the cross sectional width of each scaffold unit is approximately threefold smaller than in Fig. 3 C1–C6 when synthesized at high current density (0.4 A/cm^2^), higher temperature (670 °C) and with more lithium carbonate (50%) in the electrolyte.

We observe in Figs. 3 and 8 that molten carbonate electrolysis is conducive to a distinctive carbon nano-scaffold product in electrolytes containing 30 to 70 wt% Na_2_CO_3_ or 30 to 50 wt% K_2_CO_3_ at 670 through 750 °C. The conventional commercial path synthesis of carbon nanomaterials is by CVD, which is expensive, energy intensive and carries a high carbon footprint^42^. This is analogous to the commercial molten electrolysis of another oxide (bauxite to aluminum) is an inexpensive process, and would provide an inexpensive pathway to convert CO_2_ to a new and unusual, high surface area graphene product provides an incentive to consume CO_2_ to mitigate climate change.

In the future theoretical and experimental comparisons of the mechanism of growth differentiating carbon nano-scaffold from nanotube growth will be of interest. For now, we point out that carbon nanotubes are thermodynamically more stable (stronger, and with a closer-inter-knit rolled or spherical graphene matrix) than carbon nano-scaffolds. However, nanotube growth in molten carbonate is electrocatalytically facilitated by transition metal nucleation and when that nucleation is inhibited and fascinating alternative carbon nano morphologies are observed to occur. Three electrolysis conditions are shown here to inhibit CNT nucleation and promote growth of carbon nano-scaffolds in the presence of the transition metal nucleation metals, such as Ni, Cr and Fe. The conditions are (1) a decrease in the electrolysis temperature, (2) a decrease in the concentration of lithium in the molten carbonate electrolyte, and with decreased lithium concentration even at higher temperatures, conditions of (3) higher electrolysis current density. Consistent with these observations, are the mechanistic implications inhibiting nucleation that (1) a decrease in temperature will decrease the rate of carbonate mass transport to the nucleation for reduction. (2a) Nucleating metals such as iron had been observed to be less soluble in binary carbonates than in pure lithium carbonate^49^. (2b) A larger cation than lithium will face a larger energy barrier, when permeating the nucleation site and growing CNT walls. Similarly, (3) the greater mass transport required at higher current density will favor the 2D diffusion consistent with the scaffold’s largely planar growth, rather than the point source diffusion consistent with a nucleation point growth process.

## Conclusion

A new product, carbon nano-scaffolds with a distinctive morphology is obtained by molten carbonate electrolysis, and is controlled by specific electrolysis conditions. Unlike other carbon nano-scaffold scaffold syntheses this is product electrochemically, without a template and consists of asymmetric 20 to 100 nm thick flat multilayer graphene platelets 2 to 20 μm long oriented in a 3D neoplasticism-like geometry. We previously observed that transition metals can nucleate CNT growth during molten carbonate electrolytes. Additionally, our previous successful synthesis of carbon nano-onions, was accomplished by the avoidance of transition metals used to prevent competitive growth of the alternative CNT product. Here, transition metal ions are permitted, for example as introduced by the anode. However, the successful molten carbonate CO_2_ electrolysis to a CNS product occurs by conducting the electrolysis in electrolytic conditions not conducive to CNT formation consisting of electrolytes with decreased lithium concentration, and either at lower temperatures or at higher electrolysis current density. Electrolyte conditions of 50 wt% sodium carbonate relative to lithium carbonate at an electrolysis temperature of 670 °C produced over 80% of the CNS desired product at 85% faradaic efficiency with a brass cathode and a nickel alloy anode. This new method provides a novel, inexpensive method to remove CO_2_ and potentially turn it from a greenhouse gas pollutant into a useful, unusual carbon morphology product.
